# Explorative Laparotomy

**Published:** 1886-02

**Authors:** George R. Fowler


### Selections.

### Explorative Laparotomy.*

By Dr. George R. Fowler.

An attempt to analyze the methods of different operators, with a view of determining the conditions essential to success, discloses the fact of the existence of considerable diversity of opinion regarding these conditions. It is not incompatible with our inquiry into the advantages and merits of explorative laparotomy to consider the general subject of abdominal section, and whatever may influence, in the remotest degree, its success and failure, for the two are so indissolubly blended that whatever contributes to the well-being of the patient in the one case cannot but be of advantage in the other. In pursuing our study of the subject, it will not be possible, in the space of time allotted me, to enter deeply into all the peculiar methods ascribed to different operators, or to illustrate the different standpoints from which individual operators view the essentials of success, and the importance attached by each to any particular measure. We will consider, however, for a moment, the question of antisepsis in its relation to laparotomy in the hands of operators who achieve, in this operation, about the same general average of success year after year. In order to facilitate the inquiry, it will be convenient to divide these operators into three classes: First, those who may be said to maintain in and about the operation but a relative cleanliness; second, those who make a point of preserving the most rigid cleanliness—so rigid, indeed, as to be quite analogous to antisepsis itself; and, third, those who practice antisepsis more or less complete in detail. Why, then, it may be asked, is it, with such a difference in respect to a feature of the operation, which, judging from its importance now generally conceded in general surgery, would seem of the

*Read before the Medical Society, County of Kings.

greatest importance here, that these operators meet with almost, if not quite, equally good results? It is not easy to formulate an answer to this question, but, in my judgment, we must look far beyond any one special feature of the operation as the key-stone to success, this probably holding good to a greater extent in abdominal section than in operations involving other parts of the body. And I further believe that what an operator loses by want of attention to one particular feature, he makes up for by attention to other details upon which he lays more stress. Keith, by his most patient and careful cleansing and closing of the abdominal cavity, leaves but little chance for a septic condition to occur afterward. His exceedingly delicate manipulation and treatment of every tissue involved in the operation are, likewise, features noted and commented upon by those who have been fortunate enough to witness his operations. He, perhaps, more than any other operator, can afford to forego the employment of antiseptic precautions in abdominal surgery. Lawson Tait, who uses neither antisepsis nor even absolute cleanliness in the sense that Hegar is said to, but who, on the other hand, operates with such admirable celerity, and whose every movement is magician-like in its quickness and certainty, challenging the admiration, by these and his many ingenious and time-saving devices, of those who have made a pilgrimage to the field of his labors; exposing, as he does, the peritoneal surfaces as little as possible, and never allowing, save in the direst need, a hand other than his own to touch the parts involved in the operation; surrounded by a staff of nurses of his own training, and he a most exacting disciplinarian; and possessed of a self-confidence born of an immense experience —what need has such an operator of antisepsis, when he has a record but little short of perfection? He simply dips his hands and instruments in water from the tap, dons an old blue-cloth blouse, which, from its appearance, looks as if it might have been worn by him at every operation since the occasion of his maiden effort in surgery. From the moment he takes the scalpel in his hand, he enjoins the most perfect silence on the part of those about him; this latter he insists upon, in order that nothing may distract his attention for a single moment from the work before him. Hegar, again, may be cited as an instance of those who pursue a most stringent course of cleanliness in the same line of work, and who, it is said, with his drilled assistants, rendered almost absolutely perfect in point of purity by bathing, and fresh, clean linen at each operation, and taking every possible precaution against infection, except the use of germicides, manages to bring enough other advantages to his aid to enable him to keep the mortality of abdominal surgery, in his hands, within very narrow limits. And, lastly, it cannot be for a moment doubted, in spite of the above facts, that antisepsis has a place in abdominal surgery as elsewhere; but it must be acknowledged that its influence has not been so decidedly felt there as in other fields. Nevertheless, there are a large number of surgeons who find it of advantage to employ germicides and antiseptics in abdominal surgery. It may be, perhaps, they do not possess the painstaking methods and delicate manipulation of a Keith, nor the magical dexterity and immense experience of a Lawson Tait, and may consider the iron regimen of a Hegar more difficult to follow than antisepsis itself. These surgeons are called upon, however, and needs must, upon occasion, perform operations involving the abdominal cavity; who, not wanting in ordinary dexterity and skill, yet not phenomenal in these attributes, still manage, by dint of care and a strict adherence to the principles of antiseptic surgery, to make as good showing as their more fortunately-placed and better-known brethren. I do not wish to be understood as suggesting that the germicide bath in which the instruments are placed can compensate for want of skill in the wielding thereof; that the spray of carbolic acid solution playing over the site of operation will justify rough manipulation; nor, that the results of gross carelessness and bungling manœuvres of an incompetent operator can be hidden away, never to be heard of again, beneath the folds of an elaborate gauze and Mackintosh dressing. Far from it. Success in the surgery of the abdomen, other things being equal, seems to consist in and depend upon carrying to definite lengths carefully-matured plans and purposes. Failure may result, not from a disregard for this, that, or the other special feature of the operative procedure, but from an absence of a sufficient number of redeeming or compensating circumstances to make up the sum-total constituting success.

In the study of the subject of explorative laparotomy, it will be convenient for us to divide the cases calling for its performance into four classes, as follows: 1. Cases in which a diagnosis cannot be made without opening the abdomen and exposing the parts to the direct touch, or even, perhaps, to the sight of the surgeon, but in which further interference is thereby shown to be either impracticable or uncalled for. 2. Cases in which a provisional diagnosis only can be made, unaided by abdominal incision, and in which but slight additional risk is incurred by an immediate and radically curative procedure, based upon the knowledge thus gained. 3. Cases in which a diagnosis has been made, but in which doubt exists as to the practicability of performing a radical operation; and cases in which the choice of the particular operation best adapted to the individual case must be decided upon after incision and exploration. 4. Cases in which the patient's life is in imminent peril, and in which it becomes imperatively necessary to at once locate the lesion threatening life, and to be prepared to act promptly upon the knowledge gained by opening the abdominal cavity.—*N. Y. Medical Journal.*

